# The intricate dance of non-coding RNAs in myasthenia gravis pathogenesis and treatment

**DOI:** 10.3389/fimmu.2024.1342213

**Published:** 2024-03-27

**Authors:** Benqiao Wang, Ying Zhu, Dan Liu, Chunxiang Hu, Ruixia Zhu

**Affiliations:** Department of Neurology, The First Affiliated Hospital of China Medical University, Shenyang, China

**Keywords:** myasthenia gravis, non-coding RNAs, pathogenesis, treatment, biomarker

## Abstract

Myasthenia gravis (MG) stands as a perplexing autoimmune disorder affecting the neuromuscular junction, driven by a multitude of antibodies targeting postsynaptic elements. However, the mystery of MG pathogenesis has yet to be completely uncovered, and its heterogeneity also challenges diagnosis and treatment. Growing evidence shows the differential expression of non-coding RNAs (ncRNAs) in MG has played an essential role in the development of MG in recent years. Remarkably, these aberrantly expressed ncRNAs exhibit distinct profiles within diverse clinical subgroups and among patients harboring various antibody types. Furthermore, they have been implicated in orchestrating the production of inflammatory cytokines, perturbing the equilibrium of T helper 1 cells (Th1), T helper 17 cells (Th17), and regulatory T cells (Tregs), and inciting B cells to generate antibodies. Studies have elucidated that certain ncRNAs mirror the clinical severity of MG, while others may hold therapeutic significance, showcasing a propensity to return to normal levels following appropriate treatments or potentially foretelling the responsiveness to immunosuppressive therapies. Notably, the intricate interplay among these ncRNAs does not follow a linear trajectory but rather assembles into a complex network, with competing endogenous RNA (ceRNA) emerging as a prominent hub in some cases. This comprehensive review consolidates the landscape of dysregulated ncRNAs in MG, briefly delineating their pivotal role in MG pathogenesis. Furthermore, it explores their promise as prospective biomarkers, aiding in the elucidation of disease subtypes, assessment of disease severity, monitoring therapeutic responses, and as novel therapeutic targets.

## 1 Introduction

Myasthenia gravis (MG) is an autoimmune disorder affecting the neuromuscular junction, primarily instigated by the presence of antibodies targeting various postsynaptic components (1). Among these antibodies, anti-acetylcholine receptor antibodies (AChR-Ab) stand as the most prevalent, while antibodies against MuSK (MuSK-Ab), Lrp4, and agrin are comparatively less common (2). Additionally, biomarkers such as ColQ, Kv1.4, titin, and RyR are indeed valuable, albeit their specific pathogenic roles remain uncertain (3). The hallmark feature of MG is fatigable muscle weakness, accentuated post-exertion and relieved during rest (4). MG classification encompasses subgroups based on antibody profiles, clinical manifestations, age of onset, and thymus pathology (2). Notably, thymus involvement plays a pivotal role in AChR-Ab-positive MG (AChR-MG), with hyperplasia observed in early-onset MG (EOMG) and atrophy in late-onset MG (LOMG) (2). Thymoma-associated MG (TAMG) represents a distinct subgroup (2). The advent of high-throughput sequencing technology has redefined our perception of ncRNAs, once considered “junk DNA,” as critical cellular regulators (5). This vast class of genomic elements is typically categorized into two main groups: small or short non-coding RNAs and long non-coding RNAs (lncRNAs), based on whether their length exceeds 200 nucleotides (6). Additionally, circular RNAs (circRNAs), unique single-stranded RNA molecules formed by covalent closure at the 5’ and 3’ ends, have emerged as noteworthy constituents of ncRNAs (7). Intriguingly, ncRNAs have exhibited profound associations with diverse diseases, spanning cancer, cardiovascular and cerebrovascular conditions, as well as metabolic and autoimmune disorders (8–10). In systemic lupus erythematosus (SLE), some miRNAs, such as miR-125a, miR-125b, miR-21, miR-148a, miR,223, and miR-31, expressed abnormally, others, like miR-7, miR-155, miR-146, and miR-182 were reported to regulate B cells, T cells, or the formation of germinal centers (GCs) (11–13). While in rheumatoid arthritis (RA), miR-146a, miR-155, miR-22, and miR-10a-5p were related to the inflammatory environment (13). Let-7g-5p and NEAT1, a lncRNA, were proved to promote the proliferation of Th17 (14). With this backdrop, the investigation of ncRNAs on MG pathogenesis has become a compelling area of study. Despite significant advancements in MG management, several challenges persist (2). The etiological “triggers,” underlying molecular mechanisms, and regulatory factors at the molecular level remain elusive, confounding researchers (15). Furthermore, a subset of MG patients fails to achieve remission or substantial clinical improvement with current immunotherapies (16), a concern exacerbated by the drawbacks of long-term immunosuppressant usage, including intolerance, delayed therapeutic onset, and systemic toxicity (17). Our conviction lies in the potential of precision-targeted immunotherapy as a promising avenue to tackle these challenges. In this review, we delve into the role of ncRNAs in MG pathogenesis and explore the significance of ncRNAs in MG treatment and therapeutic monitoring.

## 2 miRNAs in MG

Among the diverse categories of small non-coding RNAs, microRNAs (miRNAs) supreme as one of the most prominent classes, distinguished by their sheer number and prevalence in research (18). These endogenous single-stranded molecules typically comprise approximately 22 nucleotides (19) and were initially discovered by Lee et al. in the early 1990s (20). In the realm of immune system modulation, miRNAs play an integral role, which explains why disruptions in their function have been associated with a range of autoimmune conditions, such as MG (21, 22) (**Table 1**).

**Table 1:** Differential expressed miRNAs in MG.

miRNA	Sample	Subtype	Antibodies	Expression	Target	Ref
let-7a-5p	serum	MG	MuSK-Ab	↓		(23)
let-7c	PBMC	MG		↓	IL-10	(24)
let-7f-5p	serum	MG	MuSK-Ab	↓		(23)
miR-106a-5p	serum	GMG, OMG		↓ (GMG<OMG)		(25)
miR-106b-5p	PBMC	MG	MuSK-Ab	↓		(26)
miR-122	serum	LOMG	AChR-Ab	↓		(27)
miR-125a-5p	thymus	EOMG	AChR-Ab	↑		(28)
miR-125a-5p	thymus	TAMG		↑	foxp3	(29)
miR-126	serum	MG	AChR-Ab	↓		(30)
miR-139-5p	thymus	GMG	AChR-Ab	↓	RGS13	(31)
miR-140-3p	serum	LOMG	AChR-Ab	↓		(27)
miR-145	PBMC	MG	AChR-Ab	↓	CD28 and NFATc1	(32)
miR-146a	PBMC	EOMG	AChR-Ab	↑		(33)
miR-146a	PBMC	EOMG	AChR-Ab	↑	IRAK1, c-REL, TRAF6, ICOS, and FAS	(34)
miR-146a	thymus, serum	EOMG	AChR-Ab	↓	IRAK1, c-REL, TRAF6, ICOS, and FAS	(34)
miR-146a	serum	MG	AChR-Ab	↑	TRAF6	(35)
miR-146a	B cells	EAMG	AChR-Ab	↑		(36)
miR-150	PBMC	EOMG	AChR-Ab	↓	MYB, P53 and AIFM2	(37)
miR-150	thymus, serum	EOMG	AChR-Ab	↑	MYB, P53 and AIFM2	(37)
miR-150-5p	serum	EOMG	AChR-Ab	↑		(38)
miR-150-5p	serum	LOMG	AChR-Ab	↑ (GMG>OMG)		(39)
miR-150-5p	serum	refractory GMG	AChR-Ab	↑		(40)
miR-150-5p	serum	EOMG, LOMG	AChR-Ab AND MuSK-Ab	↑		(41)
miR-150-5p	serum	LOMG	AChR-Ab AND Seronegative	↑ (GMG>OMG)		(42)
miR-150-5p	serum	MG	AChR-Ab AND Seronegative	↑		(43)
miR-151a-3p	serum	MG	MuSK-Ab	↓		(23)
miR-155	PBMC	MG	AChR-Ab	↑	BAFF-R/ TRAF3/NIK/NF-ĸB	(44)
miR-15a-3p	PBMC	MG	MuSK-Ab	↓		(26)
miR-15b	serum	EOMG, LOMG, TAMG	AChR-Ab	↓		(27)
miR-15b	serum	EOMG, LOMG, TAMG	AChR-Ab	↓	IL-15	(28)
miR-181a	PBMC	MG		↓	TRIM9	(45)
miR-181c	PBMC	GMG, OMG	AChR-Ab	↓ (GMG<OMG)	IL-7	(46)
miR-185	serum	LOMG	AChR-Ab	↓		(27)
miR-192	serum	EOMG	AChR-Ab	↓		(27)
miR-1930-5p	omohyoid muscles	EAMG	MuSK-Ab	↑		(47)
miR-1933-3p	omohyoid muscles	EAMG	MuSK-Ab	↑	Mrpl27 and Impa1	(47)
miR-19b-5p	thymus	TAMG		↑	TSLP	(48)
miR-20b	serum	EOMG	AChR-Ab	↓		(27)
miR-20b	serum	EOMG	AChR-Ab	↓ (GMG<OMG)	IL-8 and IL-25	(49)
miR-20b	thymoma tissues and serum	TAMG		↓	NFAT5 and CAMTA1	(50)
miR-210-3p	serum	MG	MuSK-Ab	↓		(51)
miR-21	serum	MG	AChR-Ab	↑		(30)
miR-21-5p	serum	EOMG	AChR-Ab	↑		(38)
miR-21-5p	serum	LOMG	AChR-Ab AND Seronegative	↑ (GMG>OMG)		(42)
miR-21-5p	serum	MG	AChR-Ab AND Seronegative	↑		(43)
miR-27a-3p	serum	EOMG	AChR-Ab	↓		(38)
miR-27a-3p	PBMC	MG	MuSK-Ab	↓		(26)
miR-29	thymus	EOMG	AChR-Ab	↓		(52)
miR-30e-5p	serum	LOMG	AChR-Ab	↑ (GMG>OMG)		(39)
miR-30e-5p	serum	EOMG, LOMG	AChR-Ab AND MuSK-Ab	↑		(41)
miR-30e-5p	serum	LOMG	AChR-Ab AND Seronegative	↑		(42)
miR-320a	PBMC	MG		↓	MAPK1	(54)
miR-324-3p	serum	MG	MuSK-Ab	↓		(51)
miR-340-5p	PBMC	MG	MuSK-Ab	↓		(26)
miR-3651	PBMC	EOMG	AChR-Ab	↑		(53)
miR-3654	PBMC	EOMG	AChR-Ab	↑		(53)
miR-423-5p	serum	MG	MuSK-Ab	↓		(23)
miR-452-5p	thymus	GMG	AChR-Ab	↓	RGS13	(31)
miR-522-3p	thymoma tissue, serum, Jurkat cells and CD4+ T cells	TAMG, MG without thymoma		↓ (TAMG<MG without thymoma)	SLC31A1	(54)
miR-548k	thymus	MG		↓	CXCL13	(55)
miR-612	PBMC	EOMG	AChR-Ab	↑		(53)
miR-7-5p	thymus	EOMG	AChR-Ab	↓	CCL21	(28)
miR-885-5p	serum	LOMG	AChR-Ab	↓		(27)

↓ means down regulated. ↑ means upregulated.

### 2.1 miRNAs involved in MG pathogenesis

**Figure 1:**
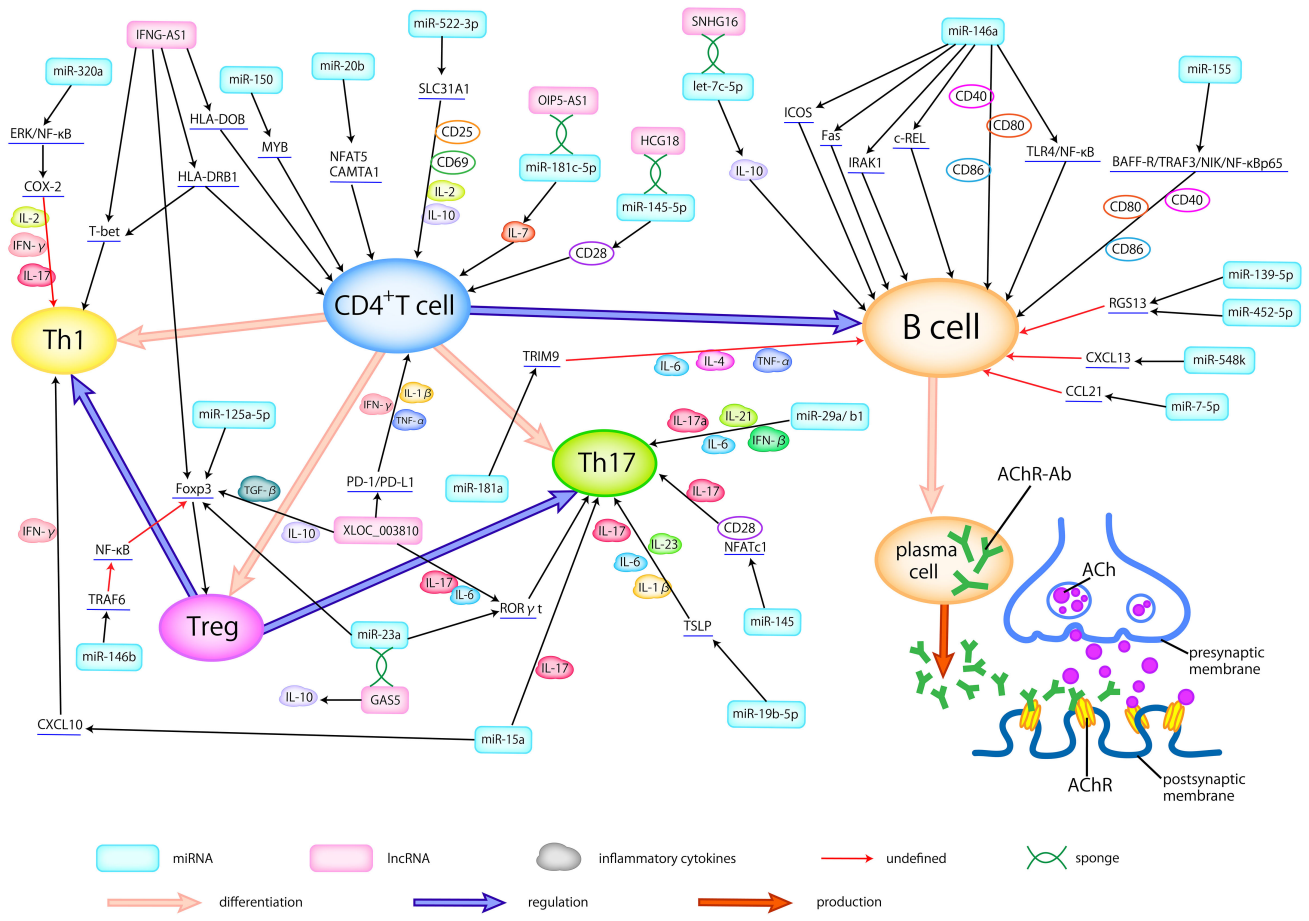
Dysregulated ncRNAs in MG pathogenesis. Abnormally expressed ncRNAs in different types of samples actively participate in the pathogenesis of AChR-MG by regulating the differentiation of T cells, broking the balance between helper T cells (Th1 and Th17) and Treg cells, activating the B cells, modulating the production of inflammatory cytokines, thus promoting the production of antibodies. Here, we delineate the regulatory relationship between abnormally expressed ncRNAs and immune cells, cytokines, and related signaling pathways.

In patients with MG, a series of dysfunctions occur at the neuromuscular junction (NMJ), driven by T cells and mediated by B cells, resulting in a complex pathogenic process (24). This process involves an array of miRNAs and proinflammatory cytokines, potentially contributing to the development of MG (**Figure 1**).

Several studies have explored the role of dysregulated miRNAs in MG by compiling a catalog of genes associated with MG susceptibility based on existing literature (27, 28, 53, 56). Yang et al. (28) conducted an analysis of 93 miRNAs associated with MG risk pathways, shedding light on their regulatory role within these pathways. Their work underscored the potential significance of some specific miRNAs in MG pathogenesis, including miR-497, miR-15a, miR-15b, miR-16, and miR-195. Cao et al. (56) pinpointed miRNA-146a as an important contributor to the regulation of numerous MG risk pathways, underscoring its pivotal role in MG pathogenesis. Moreover, in the study of Bo et al. (53), 13 dysregulated miRNAs, including miR-29b-3p, miR-145-5p, and miR-451a, were believed to play a role in MG pathogenesis. Among these risk-related miRNAs, miR-145-5p was the sole miRNA found to exhibit differential expression, suggesting its potential pivotal function in MG. In a complementary study, Qian et al. (27) reported 30 abnormally expressed miRNAs, which may participate in MG development by modulating Tregs. Collectively, these identified miRNAs offer valuable insights and may serve as guiding principles for future research endeavors aimed at unraveling the intricate pathogenesis of MG.

#### 2.1.1 Modulation of Tregs, Th17 and Th1

It is well-established that the initiation of MG critically hinges on the activation of autoreactive T cells (57). Consequently, the miRNAs exhibiting abnormal expression and responsibility for T cell proliferation and activation may represent a pivotal link in MG pathogenesis. Certain miRNAs have been identified to be involved in the regulation of T cells in MG. Cron et al. (58) discovered that miR-150 could target MYB, a proto-oncogene, thus affecting the survival of CD4^+^ and CD8^+^T cells, which may contribute to the maintenance of an immunologically activated state. Research showed that miR-20b would inhibit T cell proliferation and activation by suppressing the NFAT signaling pathway through downregulating NFAT5 and CAMTA1 (33). Moreover, decreased miR-522-3p can lead to the overexpression of CD25, CD69, IL-2, and IL-10 by targeting SLC31A1, consequently promoting the activation and proliferation of Jurkat cells, a T cell leukemia line (31). Dysregulation of key immune cell populations, including Tregs, Th17, and Th1, exerts a significant influence on MG pathogenesis. Studies have reported that the expression of Tregs is associated with disease severity, with observations of decreased Foxp3, a Treg-related cytokine, in peripheral blood mononuclear cells (PBMCs) of MG patients (39). In an AIRE knockout mouse model, reduced Tregs and increased Th17 cells in the thymus rendered the mice more susceptible to experimental autoimmune myasthenia gravis (EAMG) (42), mirroring the pathogenesis of LOMG and TAMG (15). Villegas et al. (46) noted that an imbalance between Tregs and Th17 cells is associated with chronic thymic inflammatory conditions in AChR-MG.

The lapses in Treg function result in the loss of their inhibitory effect on excessive inflammatory responses, leading to uncontrolled inflammatory cell activity in MG (25). This unchecked immune activity gives rise to chronic inflammation and thymic hyperplasia, further disrupting immune tolerance mechanisms. Recent research outcomes have unveiled a miR-146b-TRAF6-NF-κB-Foxp3 pathway, which plays a crucial role in suppressing Treg proliferation and its inhibitory function (49, 59). Intriguingly, Yan et al. (60) proposed that overexpressed miR-146a may contribute to the pathogenesis of MG by promoting TRAF6 expression, which could further disrupt Treg function. miR-125a-5p was confirmed to negatively regulate Foxp3, consequently inhibiting the generation of Treg with a great possibility (37). Additionally, there is a positive correlation between the reduced miR-126 and Foxp3 mRNA in MG, providing strong evidence for the downregulation of Treg activity (50). In summary, differentially expressed miRNAs in MG have been observed to inhibit Treg proliferation and weaken their inhibitory function. Consequently, these miRNA-mediated changes impair immunological tolerance mechanisms and play an important role in the pathogenesis of MG.

Compelling scholarly evidence has demonstrated that the activation of Th17 and Th1 cells, when losing the control of Tregs, can lead to the release of pro-inflammatory cytokines such as INF-γ and IL-17 (61), which may build the bridge between some dysregulated miRNAs and MG. For instance, Wang et al. (62) revealed that miR-145 could serve as a regulator that promotes T cell proliferation and differentiation into Th17 cells, by targeting CD28 and NFATc1. Similarly, Cron et al. (34) reported a reduction of the miR-29 family within thymic tissues of MG, potentially in connection with an increase of IFN-β. Among these, miR-29a/b1, part of a miR-29a genomic cluster, could possibly facilitate the flourishing of Th17 cells. Besides, miR-19b-5p in TAMG has been shown to post-transcriptionally inhibit thymic stromal lymphopoietin (TSLP), thereby regulating Th17 cells and their related cytokines (44). It’s reasonable to speculate that these abnormally expressed miRNAs may function as regulators, promoting the release of Th17-related cytokines (IL-6, TGF-β, IL-17, IL-1β, and IL-23) and the proliferation of Th17 cells. On the grounds of research by Cheng et al. (45), with the target of MAPK1, miR-320a may influence the Th1-associated cytokines, like IL-2, and IFN-γ, by adjusting COX-2 through the regulation of ERK/NF-κB pathways. In addition, several miRNAs contribute to the pathogenesis of MG by influencing both Th1 and Th17 responses concurrently. Liu et al. (63) reported that miR-15a could affect the CXCL10 gene by regulating Th1- and Th17-related cytokines to generate immune responses. In conclusion, the dysregulation of miRNAs can disrupt Treg function, promote the proliferation of Th1 and Th17 cells, and result in the overexpression of pro-inflammatory cytokines, thus modulating the pathogenesis of MG.

#### 2.1.2 miRNAs as regulators of B cell activation

B cells, integral players in the pathogenesis of MG, participate in the formation of ectopic GCs, receive signals from antigen-presenting cells (APCs), and contribute to the production of autoimmune antibodies (38). Many dysregulated miRNAs can modulate chemokines and specific signaling pathways to promote the maturation of autoreactive B cells and the development of GCs.

miR-155, found to be elevated in B cells, engaged in the immune response through the BAFF-R/TRAF3/NIK/NF-κB p65 pathway, mediating the survival of activated B cells and increasing the production of AChR antibodies by regulating co-stimulatory molecules (48). The binding of BAFF to BAFF-R is necessary for B cell maturation and survival (43). And miR-155 can promote the expression of BAFF-R and TRAF3 to facilitate the phosphorylation of NIK, thus helping the NF-κB to translocate into the nuclear of B cells (48). At the same time, miR-155 can also modulate the co-stimulatory molecules, such as CD40, CD80, and CD86, to enhance the production of antibodies by B cells. Two independent studies mentioned an elevation of miR-146a in AchR-specific B cells and they also reported that miR-146a could affect B cell immunity (64, 65). Notably, one of these studies proposed that miR-146a may facilitate the activation of B cells and the production of antibodies by modulating the TLR4 and NF-κB pathways (64). In a study by Bortone et al. (66), the downregulated miR-146a showed a noteworthy negative correlation with IRAK1, c-REL, ICOS, and FAS in the follicular hyperplastic thymus of EOMG. The genes targeted by miR-146a appear to collectively contribute to the activation of B cells and the formation of GCs. Firstly, the downregulation of miR-146a allows IRAK1 to induce excessive inflammation and disrupt immune tolerance through the TLR signaling pathway. Secondly, the deficiency of miR-146a can enhance the activation of c-REL, thereby promoting the proliferation and differentiation of B cells and amplifying GCs formation. Furthermore, reduced miR-146a may fail to effectively restrict the aggregation of follicular helper T cells (Tfhs) and the proliferation of B cells in GCs by targeting ICOS. Lastly, miR-146a can downregulate FAS expression, promoting lymphoproliferation and GCs formation. Wang et al. (67) found that miR-181a may influence the levels of inflammatory cytokines, such as TNF-α, IL-4, and IL-6, affecting B cell proliferation by regulating TRIM9.

Additionally, miR-548k, reduced in the thymus of MG, can target CXCL13 (40), which plays a role in directing B cells and facilitating GCs formation. miR-452-5p and miR-139-5p were associated with the promotion of RGS13 expression, leading to B cell proliferation and GCs expansion (54). miR-150-5p has shown a positive correlation with CD19^+^ and CD27^+^ B cells, suggesting its involvement in B cell differentiation, memory B cell formation, and further possible immune responses, including B cell activation and antibody production (30). Additionally, the abnormally expressed miR-126 and miR-21 in PBMCs were proved to upregulate the expression of IL-6, thus promoting the proliferation and differentiation of B cells, consequently facilitating the production of antibodies (50). let-7c was reported to target IL-10, probably participating in MG pathogenesis by stimulating B cells (68). Besides, the reduced miR-7 in thymus was reported to upregulate the expression of CCL21, thus helping the formation of GCs (69).

### 2.2 miRNAs as clinical biomarkers and therapeutic targets

#### 2.2.1 miRNAs engaged in AChR-MG

AChR-Ab is the most prevalent antibody in MG, accounting for approximately 85% of cases (70). AChR-MG can be further classified according to clinical features. Interestingly, there are some differences in thymus pathology among EOMG, LOMG and TAMG. EOMG refers to individuals under the age of 50, characterized by hyperplastic thymus pathology. In contrast, LOMG manifests in individuals over the age of 50, commonly associated with thymic atrophy. TAMG, on the other hand, represents a paraneoplastic syndrome of thymoma (4). A few of studies (54, 55, 58, 64, 66, 69, 71) revealed some differentially expressed miRNAs, such as miR-139-5p, miR-452-5p, miR-612, miR-3654, miR-365, miR-150, miR-20b, miR-192, miR-7, miR-125a-5p, and miR-146a, in the AChR antibody-positive EOMG (AChR-EOMG) patients. A decrease in miR-15b expression was observed in the serum of both early-onset and late-onset AChR-MG (71), which aligns with findings from an animal model of EAMG in mice injected with Torpedo AChR (72). In addition, in patients with AChR antibody-positive LOMG (AChR-LOMG), miR-122, miR-140-3p, miR-185, miR-885-5p, miR-106b-3p, miR-223-5p, miR-140-5p, miR-19b-3p, miR-30e-5p, and miR-150-5p were found to express abnormally (29, 32, 71). These findings suggest a nuanced expression profile of dysregulated miRNAs between EOMG and LOMG. Many miRNAs were reported to express aberrantly in TAMG, including miR-125a-5p, miR-19b-5p, miR-20b and miR-522-3p (25, 33, 50, 58). Additionally, a study by Shi et al. (72) indicated a decrease in miR-15b expression in TAMG, EOMG, and LOMG patients.

Several dysregulated miRNAs in AChR-MG, compared with ocular myasthenia gravis (OMG), have more severe abnormalities in generalized myasthenia gravis (GMG), some of which are correlated with quantitative myasthenia gravis scores (QMGs) or myasthenia gravis composite scores (MGCs), reflecting the severity of MG and further serving as potential biomarkers for disease surveillance with great possibility. Research findings indicated that both OMG and GMG patients showed elevated miR-150-5p, miR-21-5p, and miR-30e-5p, which had a positive correlation with MGCs and significantly more overexpressed in GMG than in OMG (29, 32). Conversely, the lower expressed miR-181a, miR-106a-5p, and miR-20b in MG, had a negative relationship between QMGs, with GMG displaying notably reduced levels compared to OMG (73–75). Additionally, several studies (51, 76) on curative effect indicated a parallel between the reduction of AChR-Ab titers and the improvement of symptoms. A fast decrease of AChR-Ab titers more than 50% also made a clinical sense (23). Interestingly, some studies showed that miR-126, and miR-145 were negatively correlated with AChR-Ab titer (50, 62), while miR-21 was positively related to AChR-Ab titer (50). We therefore speculate that specific ncRNAs may also be useful for efficacy detection.

MiR-150-5p, miR-30e-5p, and miR-146a levels in the serum of AChR-MG patients have shown promising responses to various treatments like thymectomy and immunosuppression, indicating a correlation between their levels and clinical improvement (26, 30, 32, 35, 52, 58, 66). These miRNAs could potentially serve as biomarkers for monitoring therapeutic efficacy and reflecting the disease severity. Furthermore, research by Zhang et al. (65) suggested that silencing miR-146a could reduce the expression of CD40, CD80, and CD86 on the surface of B cells, inhibit B cell differentiation, and consequently reduce the production of anti-AChR antibodies. By comparing the level of miR-146a and the expression of c-REL in the thymus of MG in corticosteroid-naïve patients and corticosteroid-treated patients, Bortone et al. (66) reported that after corticosteroid treatment, the immune response of c-REL, which was originally active in GCs and infiltrating B cells of untreated patients, was strongly inhibited, highlighting the miR-146a/c-REL axis as a potential therapeutic target for immunosuppressants. These discoveries suggests that miR-146a could serve as both a therapeutic target and a monitoring indicator for treatment efficacy. Moreover, Wang et al. (48) demonstrated that silencing miR-155 could inhibit the production of anti-T-AChR antibodies, making miR-155 another potential treatment target. Sengupta et al. (54) proposed that miR-139-5p and miR-452-5p might be considered for MG treatment, especially for EOMG, as their mimics inhibit B cell chemotaxis and GCs formation. Additionally, Cavalcante et al. reported that AChR-MG with abnormally expressed miRNAs such as miR-323b-3p, miR-409-3p, miR-485-3p, miR-181d-5p, and miR-340-3p are not sensitive to immunosuppressants (47), suggesting the presence of these miRNAs may result in poor treatment. In other words, these miRNAs may be used to forecast clinical response.

In essence, miRNAs exhibit subtle variations in expression across MG subgroups. Certain miRNAs with abnormal expression levels have the potential to facilitate disease monitoring, aid in targeted therapy, and assess treatment efficacy. Firstly, several miRNAs tend to normalize after appropriate treatment. Secondly, certain miRNAs may be implicated in antibody production, making them potential targets for MG treatment. Lastly, the presence of specific miRNAs may indicate the response to treatment. These miRNAs hold promise as specific biomarkers reflecting disease severity or treatment responses and may serve as novel targets for MG therapy. Consequently, there arises the prospect of precision therapy and personalized monitoring for individuals with MG.

#### 2.2.2 miRNAs intertwined with MuSK-MG

MuSK antibodies are present in approximately 1-10% of MG cases, predominantly affecting young women under the age of 40 (77). Although research on MuSK-Ab-positive MG (MuSK-MG) has not been as extensive as that on AChR-MG, several studies have reported differentially expressed miRNAs in this subgroup. Sabre et al. (36) observed a significant decrease in miR-210-3p and miR-324-3p levels in the serum of MuSK-MG. Punga et al. (78) identified differential expression of let-7a-5p, let-7f-5p, miR-151a-3p, and miR-423-5p in the serum of MuSK-MG compared to healthy controls. An investigation of PBMCs in MuSK-MG revealed 5 overexpressed and 96 under expressed miRNAs, with marked decreases observed in miR-340-5p, miR-106b-5p, miR-27a-3p, and miR-15a-3p (79). Moreover, an animal study identified 13 abnormally expressed miRNAs in the omohyoid muscle of MuSK-Ab-positive EAMG mice, particularly highlighting the elevation of miR-1933-3p and miR-1930-5p (80). Additionally, in a therapeutic study, the serum level of miR-151a-3p in MuSK-MG patients decreased after treatment (81), indicating a possibility for miR-151a-3p as potential therapeutic target and monitor for treatment efficacy in MuSK-MG.

Unlike AChR and MuSK antibodies, Lrp4 and agrin antibodies are rare in MG, resulting in limited research on these subgroups. Although some miRNAs have been identified as potential regulators of Lrp4 or agrin (82–88), the precise relationship between miRNAs and these two antibodies remains undiscovered (89).

## 3 lncRNAs in MG

Being different from miRNAs, lncRNA genes exhibit low conservation across evolution (90). They play a pivotal role in modulating the immune system through various mechanisms, such as guiding the development of immune cell lineages, orchestrating dynamic transcriptional programs that activate immune cells (91), and regulating immune-related genes (92). As an autoimmune disease, MG has a close connection with lncRNAs (**Table 2**).

**Table 2:** Differential expressed lncRNAs in MG.

miRNA	Sample	Subtype	antibodies	Expression	Target	Ref
A_19_P00315959	PBMC	TAMG, MG without thymoma		↑ (TAMG>MG without thymoma)		(93)
A_21_P0002844	PBMC	TAMG		↓		(93)
A_21_P0010030	PBMC	TAMG		↓		(93)
A_24_P927716	PBMC	TAMG		↑		(93)
ATP6VOE2-AS1	PBMC	MG	AChR-Ab	↓		(94)
ENSG000000218510.3	thymus	TAMG		↓		(95)
ENSG00000250850.2	PBMC	MG	AChR-Ab	↓		(94)
ENSG00000259354.1	PBMC	MG	AChR-Ab	↑		(94)
ENST00000581362.1	serum exosome	MG	AChR-Ab	↑		(96)
ENST00000583253.1	serum exosome	MG	AChR-Ab	↑		(96)
GAS5	PBMC	EOMG	AChR-Ab	↓	miR-23a	(97)
GAS5	PBMC	GMG	AChR-Ab	↓		(98)
HCG18	PBMC	MG		↑	miR-145-5p	(99)
IFNG-AS1	PBMC	MG	AChR-Ab	↓		(100)
LINC00173	PBMC	MG		↑		(107)
MALAT-1	PBMC	MG		↓	miR-338-3p	(100)
NR_022008.1	serum exosome	MG	AChR-Ab	↑		(96)
NR_046098.1	serum exosome	MG	AChR-Ab	↑		(96)
NR_104677.1	serum exosome	MG	AChR-Ab	↑		(96)
oebiotech_02627	PBMC	MG without thymoma		↓		(93)
oebiotech_03926	PBMC	MG without thymoma		↑		(93)
oebiotech_11933	PBMC	TAMG, MG without thymoma		↑		(93)
oebiotech_13222	PBMC	TAMG, MG without thymoma		↑ (TAMG>MG without thymoma)		(93)
oebiotech_16223	PBMC	TAMG, MG without thymoma		↓ (TAMG<MG without thymoma)		(93)
oebiotech_22482	PBMC	MG without thymoma		↓		(93)
oebiotech_22652	PBMC	TAMG, MG without thymoma		↓ (TAMG<MG without thymoma)		(93)
OIP5-AS1	PBMC	MG		↑	miR-181c-5p	(101)
SNHG16	PBMC	MG		↑	let-7c-5p	(102)
XLOC_000734	PBMC	MG	AChR-Ab	↓		(94)
XLOC_003810	PBMC	MG	AChR-Ab	↑		(94)
XLOC_003810	thymus	TAMG		↑		(103)
XLOC_003810	thymus, PBMC	TAMG		↑		(104)
XLOC_005780	PBMC	MG	AChR-Ab	↑		(94)
XLOC_006297	thymus	TAMG		↑		(95)

↓ means down regulated. ↑ means upregulated.

### 3.1 The role of lncRNAs in MG pathogenesis

LncRNAs have the capacity to modulate the balance between T cell subtypes and regulate proinflammatory cytokines, thereby participating in the pathogenesis of MG (**Figure 1**). Interestingly, some lncRNAs can serve as the ceRNA, certain RNA sequestering another RNA to influence its primary targets through microRNA response elements (MREs) (95) to regulate the development of MG.

Several studies have aimed to construct lncRNA-related networks to uncover potential relationships among genes, ncRNAs, and signaling pathways, thus shedding light on MG pathogenesis. For instance, Xu et al. (104) identified LINC00173, FAM13A-AS1, and OIP5-AS1 as closely associated with phosphatase and tensin homolog (PTEN), with LINC00173 showing promise as a potential MG biomarker. Another study by Lu et al. (97) suggested that lncRNAs such as NR_104677.1, NR_022008.1, and ENST00000581362.1 may play a role in MG progression by acting as miRNA sponges in specific tuples involving miRNAs like miR-15b-5p and miR-146a-5p. Hong et al. (93), in their research involving culturing human primary myoblast cells with AChR antibodies, established a co-expressed network of lncRNAs and protein-coding RNAs. They found that MEG3, RP11-184M15.1, and SNHG3 were co-expressed with several protein-coding RNAs, and MEG3, in particular, was linked to cellular homeostasis pathways. The dysregulation of MEG3 might contribute to the development of MG by disrupting cellular equilibrium.

#### 3.1.1 Regulation of immune cells and cytokines

Just like miRNAs, dysregulated lncRNAs can also influence pro-inflammatory cytokines and associated signaling pathways, promoting the proliferation of CD4+ T cells and B cells, inhibiting the proliferation of Treg cells, and activating Th17 and Th1 cells to disrupt immune homeostasis in MG.

In the study by Xu et al. (98), GAS5 was found to decrease in CD4^+^T cells and directly negatively regulate the expression of miR-23a. Furthermore, overexpressed GAS5 was shown to disrupt the balance between Th17 and Treg cells, restraining Th17 differentiation by sponging miR-23a. Another related study (103) indicated that the upregulation of GAS5 was associated with increased levels of IL-10, coinciding with an improvement in MG symptoms. Two studies by Hu et al. (94, 102) demonstrated that XLOC_003810 was elevated in thymic CD4^+^T cells in MG. In one study, overexpression of XLOC_003810 in TAMG disrupted the balance between Treg and Th17 by favoring Th17 differentiation and increasing Th17-associated markers such as RORγt, IL-6, and IL-17, while reducing Treg-related markers like Foxp3, TGF-β1, and IL-10. Another study emphasized that XLOC_003810 promoted the expression of CD4^+^T cells and their inflammatory cytokines, such as IFN-γ, TNF-α, and IL-1β, highlighting its significant role in MG pathogenesis, especially in TAMG. Luo et al. (100) found lower levels of IFNG-AS1 in PBMCs of MG, which were negatively correlated with HLA-DOB and HLA-DRB1. They also demonstrated that increased IFNG-AS1 could suppress the proliferation of Th1 cells and promote the expansion of Treg cells, along with some of their transcription factors. IFNG-AS1 was considered to downregulate CD40L and T-bet in CD4^+^T cells of MG, partly dependent on HLA-DRB1, implying the involvement of IFNG-AS1 in CD4^+^ T-related immune responses. HCG18, an upregulated ceRNA in PBMCs, sponging miR-145-5p to modulate CD28, was proved to inhibit apoptosis and enhance the proliferation of Jurkat cells (99). Moreover, OIP5-AS1 was also verified to have a similar effect on Jurkat cells in another study (105), which can be achieved through regulating IL-7 by sponging miR-181c-5p. Additionally, Wang et al. (101) demonstrated that the upregulated SNHG16 can serve as a ceRNA, competitively binding with let-7c-5p in PBMCs of MG. This action not only facilitates Jurkat cell proliferation and inhibits their apoptosis but also influences the level of IL-10, potentially further promoting the activation of B cells.

### 3.2 Clinical prospects and therapeutic potential of lncRNAs

In AChR-MG, some of the abnormally expressed lncRNAs are closely related to QMGs and MG Impairment Index (MGII), making them indicative of disease severity, while others may aid in subgroup diagnosis, particularly for TAMG. Luo et al. (100) found that IFNG-AS1 in PBMCs exhibited significant negative correlations with QMGs. Besides, GAS5 was also observed to have strong associations with QMGs and MGII (103). In another expression profile, five lncRNAs, NR_104677.1, ENST00000583253.1, NR_046098.1, NR_022008.1, and ENST00000581362.1, were reported to remarkedly overexpress in MG exosome, wherein, NR_046098.1 was upregulated prominently with the severity of MG (97). Another study profiling lncRNA expression identified numerous dysregulated lncRNAs in PBMCs (96). Among them, ENSG00000250850.2, ATP6VOE2-AS1, and XLOC_000734 were the three most significantly downregulated lncRNAs, while XLOC_003810, XLOC_005780, and ENSG00000259354.1 were the top three highly upregulated lncRNAs when compared to healthy controls. In the context of TAMG, there were 3,699 upregulated lncRNAs and 661 downregulated lncRNAs identified. Notably, XLOC_006297 exhibited the highest expression, while ENSG000000218510.3 had the lowest expression levels among the identified lncRNAs (106). In a study involving 34 MG patients and 13 healthy controls, Luo et al. (107) identified significant dysregulation of lncRNAs in PBMCs. Their findings revealed distinct expression patterns when comparing different experimental and control groups. Some of their key observations relied on the fact that, compared with the control group, oebiotech_11933 and A_24_P927716 were the top two overexpressed lncRNAs, while A_21_P0010030 and A_21_P0002844 were the least expressed in TAMG patients. They have also verified that the expression of A_19_P00315959 and oebiotech_13222 in TAMG was much more elevated than that in non-thymoma MG, while the level of oebiotech_22652 and oebiotech_16223 in TAMG patients was much lower. Finally, oebiotech_11933 and oebiotech_03926 were significantly upregulated, whereas ebiotech_02627 and oebiotech_22482 were substantially reduced in MG patients without thymoma versus healthy controls.

In addition to serving as biomarkers, the dysregulated lncRNAs also have the potential to be targeted for treatment. Kong et al. (108) suggested that MALAT-1 may function as an endogenous sponge, competing with male-specific lethal 2 (MSL2) to bind miR-338-3p. This interaction could lead to the inhibition of T cells and potentially play a protective role, making the MALAT-1-miR-338-3p-MSL2 network a promising therapeutic target.

## 4 circRNAs in MG

circRNAs were first reported in viroids by Sanger et al. (109) in 1976. These molecules, characterized by their circular structure, can be categorized into four main types (110): exonic circRNAs (ecircRNAs), exon-intron circRNAs (EIciRNAs), intronic circRNAs (ciRNAs), and tRNA intronic circular RNAs (tricRNAs). While the functions of most circRNAs remain poorly understood, they are known to play crucial roles in immune regulation, including the adjustment of immune cells, handling immune responses, and modulating immune signaling pathways (111).

Recent research has begun to shed light on aberrant circRNAs in MG (**Table 3**), although only a limited number of studies have explored the connection between circRNAs and MG to date. These abnormally expressed circRNAs may contribute to the pathogenesis of MG, reflect disease severity, and present a potential as therapeutic targets.

**Table 3:** Differential expressed circRNAs in MG.

miRNA	Sample	Subtype	Expression	Target	Ref
circ-5333-4	serum	MG	↑		(112)
circ-FBL	serum	MG	↑	miR-133 and PAX7	(113)

↑ means upregulated.

Two published studies on circRNAs and MG have identified several circRNAs with altered expression patterns that may participate in MG pathogenesis and serve as potential biomarkers: First, four circRNAs, circ-5333-4, circ-0076490, circ-0047056, and circ-16293-1, were found to be dysregulated in the peripheral blood of MG patients. Among these, circ-5333-4 exhibited significant upregulation and was associated with QMGs, indicating a potential connection between its expression and MG severity (112). The specificity of circ-5333-4 for MG was highlighted by comparing MG and SLE cohorts. And circ-5333-4 was proposed to be part of a ceRNA regulation network involving miR-4310 and MORF4L2, warranting further investigation of potential for MG diagnosis and monitoring. Second, in a more recent study investigating circRNAs in MG serum, circ-FBL was identified as an overexpressed circRNA (113). It was suggested to function as a ceRNA by sponging miR-133, thereby promoting the expression of Pax7. This interaction was found to enhance myoblast proliferation, potentially compensating for MG-related muscle weakness, suggesting a therapeutic effect when Pax7 regulated by circ-FBL expressed to a great extent.

## 5 Conclusions and future directions

MG is characterized by complexity in diagnosis, treatment variability, and lacks curative options. ncRNAs have emerged as vital biomarkers for delineating MG subgroups, assessing severity, monitoring responses, and offering treatment targets. Research highlights aberrant ncRNAs in MG pathogenesis, suggesting their potential as biomarkers (**Table 4**). While immune dysregulation and antibody production are known MG drivers, precise disease initiators remain elusive. ncRNAs are integral in MG pathogenesis, potentially offering avenues for disease prevention. Some differentially expressed ncRNAs could modulate T cell differentiation, disrupt the delicate balance between helper T cells and regulatory T cells, activate B cells, regulate inflammatory cytokine production, and function as ceRNAs or not to construct intricate networks involving related pathways. Moreover, Losen et al. (115) reported that short hairpin RNA (shRNA) could disturb the neuromuscular transmission by reducing the level of rapsyn, a bridge protein between AChR and the cytoskeleton in the postsynaptic membrane (2). In addition, some ncRNAs were reported to protect the muscle endplate from the complement attack (116). The regulation of several miRNAs, such as miR-206, miR-127, and miR-29b, can affect the differentiation of satellite cells, the main muscle stem cells (117). A few of ncRNAs were found to serve as ceRNAs to modulate the muscle development (113, 117). It follows that ncRNAs may not only influence the production of specific antibodies, but also take part in the stabilization of postsynaptic membrane, the regulation of complement, and the regeneration of muscle.

**Table 4:** Sn, Sp, and AUC of ncRNAs as MG biomarker candidates.

ncRNAs	subtype	Sn	Sp	AUC	Ref
miR-423-5p	MuSK-MG	NA	NA	0.740	(78)
miR-340-5p	MuSK-MG	NA	NA	0.809	(79)
miR-30e-5p	EOMG, LOMG	55.60%	85.70%	0.69	(114)
miR-30e-5p	OMG, GMG	96.00%	NA	NA	(29)
miR-29c-5p	EOMG	NA	NA	0.875	(34)
miR-29b-3p	EOMG	NA	NA	0.792	(34)
miR-29a-3p	EOMG	NA	NA	0.93	(34)
miR-27a-3p	MuSK-MG	NA	NA	0.913	(79)
miR-15a-3p	MuSK-MG	NA	NA	0.936	(79)
miR-151a-3p	MuSK-MG	NA	NA	0.74	(78)
miR-150-5p	EOMG	90.00%	58.40%	0.77	(114)
miR-150-5p	EOMG, LOMG	85.20%	48.20%	0.7	(114)
miR-146a	EOMG	NA	NA	0.782	(66)
miR-146a	MG	73.20%	67.60%	0.782	(60)
miR-106b-5p	MuSK-MG	NA	NA	0.809	(79)
miR-106a-5p	OMG	NA	NA	0.728	(73)
miR-106a-5p	GMG	NA	NA	0.813	(73)
let-7f-5p	MuSK-MG	NA	NA	0.726	(78)
let-7a-5p	MuSK-MG	NA	NA	0.659	(78)
GAS5	GMG	NA	NA	0.8969	(103)
circ-5333-4	MG	84.21%	75.00%	0.7895	(112)
circ-16293-1	MG	84.21%	65.00%	0.7263	(112)
circ-0076490	MG	78.95%	65.00%	0.7816	(112)
circ-0047056	MG	55.00%	84.21%	0.6921	(112)

Sn, sensibility; Sp, specificity; AUC, area under the curve; NA, not available.

Several drugs targeting T cells and B cells have sprung up to treat MG. In parallel, the burgeoning interest in the role of ncRNAs in modulating B cells and T cells presents a fertile ground for exploration. With the potential for regulating GCs formation, B cell differentiation, certain ncRNAs, such as miR-146a, miR-155, miR-139-5p, and miR-452-5p, exhibit potential as innovative therapeutic targets, while others may serve as prognostic indicators for clinical response. Furthermore, some ncRNAs with protective roles in MG may harbor therapeutic potential, like circ-FBL (113).

Beyond their therapeutic implications, ncRNAs can also serve as indicators for monitoring clinical therapeutic responsiveness, by changing in their levels before and after the treatment and effecting on antibody titers, thus facilitating individualized treatment approaches. Notably, the presence of some miRNAs, such as miR-323b-3p, miR-409-3p, miR-485-3p, miR-181d-5p and miR-340-3p, made patients insensitive to immunosuppressive therapies (47), suggesting the need for a reevaluation of treatment strategies.

The preceding findings underscore the significance of further exploration into the role of ncRNAs in MG. However, several critical areas remain uncharted. Firstly, a subset known as ‘seronegative MG’ (41) patients that lack detectable antibodies still present a diagnostic challenge, and the exploration of ncRNAs may offer insights into the diagnosis of them. Furthermore, we know less about the ncRNAs associated with uncommon antibodies at the neuromuscular junction, such as anti-LRP4 and Agrin antibodies. Secondly, many studies have focused solely on documenting the aberrant expression of ncRNAs without delving into their specific roles in MG pathogenesis. Lastly, there is a paucity of research on circRNAs in MG, despite their potential to uncover novel aspects of MG pathogenesis and open new avenues for treatment. In summary, the enigmatic pathogenesis of MG and its association with ncRNAs necessitate comprehensive and ongoing exploration to expand our understanding. This expanded knowledge will better equip us to navigate the complexities associated with the disease and develop more tailored and effective treatment approaches.

## Author contributions

BW: Writing – review & editing, Writing – original draft. YZ: Writing – review & editing. DL: Writing – review & editing. CH: Writing – review & editing. RZ: Writing – review & editing.

## Glossary

**Table:**

AChR-Ab	anti-acetylcholine receptor antibodies
AChR-EOMG	AChR antibody-positive EOMG
AChR-LOMG	AChR antibody-positive LOMG
AChR-MG	AChR-Ab-positive MG
APCs	antigen-presenting cells
ceRNA	competing endogenous RNA
circRNAs	circular RNAs
ciRNAs	intronic circRNAs
EAMG	experimental autoimmune myasthenia gravis
ecircRNAs	exonic circRNAs
EIciRNAs	exon-intron circRNAs
EOMG	early-onset MG
GCs	germinal centers
GMG	generalized myasthenia gravis
lncRNAs	long non-coding RNAs
LOMG	late-onset MG
MG	myasthenia gravis
MGCs	myasthenia gravis composite scores
MGII	MG Impairment Index
miRNAs	microRNAs
MREs	microRNA response elements
MSL2	male-specific lethal 2
MuSK-Ab	antibodies against MuSK
MuSK-MG	MuSK-Ab-positive MG
ncRNAs	non-coding RNAs
NMJ	neuromuscular junction
OMG	ocular myasthenia gravis
PBMCs	peripheral blood mononuclear cells
PTEN	phosphatase and tensin homolog
QMGs	quantitative myasthenia gravis scores
shRNA	shRNA short hairpin RNA
SLE	systemic lupus erythematosus
TAMG	thymoma-associated MG
Tfhs	follicular helper T cells
Th1	helper 1 cells
Th17	helper 17 cells
Tregs	regulatory T cells
tricRNAs	tRNA intronic circular RNAs
TSLP	thymic stromal lymphopoietin
